# Antimicrobial Properties of Biofunctionalized Silver Nanoparticles on Clinical Isolates of *Streptococcus mutans* and Its Serotypes

**DOI:** 10.3390/nano6070136

**Published:** 2016-07-22

**Authors:** Ángel Manuel Martínez-Robles, Juan Pablo Loyola-Rodríguez, Norma Verónica Zavala-Alonso, Rita Elizabeth Martinez-Martinez, Facundo Ruiz, René Homero Lara-Castro, Alejandro Donohué-Cornejo, Simón Yobanny Reyes-López, León Francisco Espinosa-Cristóbal

**Affiliations:** 1 Graduate Program in Orthodontics, Faculty of Dentistry, Mexicali Campus, Autonomous University of Baja California, Alvaro Obregon and Julian Carrillo Avenue, Nueva, 21100 Mexicali, Baja California, México; angelorto@outlook.es; 2 Master Program in Advanced Dentistry, Faculty of Dentistry, Autonomous University of San Luis Potosi, Manuel Nava Avenue, Universitary Campus, 78290 San Luis Potosí, S. L. P., México; juanpablo.loyola8@gmail.com (J.P.L.-R.); ritae_martinez@hotmail.com (R.E.M.-M.); 3 Doctoral Program in Dental Sciences, Faculty of Dentistry, Autonomous University of San Luis Potosi, Manuel Nava Avenue, Universitary Campus 78290 San Luis Potosí, S. L. P., México; nveroza@hotmail.com; 4 Faculty of Science, Autonomous University of San Luis Potosi, Salvador Nava Avenue, 78290 San Luis Potosí, S. L. P., México; facundo@fciencias.uaslp.mx; 5 Faculty of Chemistry, Juarez University of Durango State, Chihuahua Avenue, 34120 Durango, Dgo., México; lcrh75@hotmail.com; 6 Department of Dentistry, Biomedical Science Institute, Autonomous University of Juarez City, Envolvente del PRONAF and Estocolmo Avenues, 32310 Juárez, Chihuahua, México; adonohue@uacj.mx; 7 Biomedical Science Institute, Autonomous University of Juarez City, Envolvente del PRONAF and Estocolmo Avenues, 32310 Juárez, Chihuahua, México; yobannyr@yahoo.com.mx

**Keywords:** silver nanoparticles, bovine serum albumin, chitosan, *Streptococcus mutans*, antimicrobial effect

## Abstract

(1) Background: *Streptococcus mutans* (*S. mutans*) is the principal pathogen involved in the formation of dental caries. Other systemic diseases have also been associated with specific *S. mutans* serotypes (*c*, *e*, *f*, and *k*). Silver nanoparticles (SNP) have been demonstrated to have good antibacterial effects against *S. mutans*; therefore, limited studies have evaluated the antimicrobial activity of biofunctionalized SNP on *S. mutans* serotypes. The purpose of this work was to prepare and characterize coated SNP using two different organic components and to evaluate the antimicrobial activity of SNP in clinical isolates of *S. mutans* strains and serotypes; (2) Methods: SNP with bovine serum albumin (BSA) or chitosan (CS) coatings were prepared and the physical, chemical and microbiological properties of SNP were evaluated; (3) Results: Both types of coated SNP showed antimicrobial activity against *S. mutans* bacteria and serotypes. Better inhibition was associated with smaller particles and BSA coatings; however, no significant differences were found between the different serotypes, indicating a similar sensitivity to the coated SNP; (4) Conclusion: This study concludes that BSA and CS coated SNP had good antimicrobial activity against *S. mutans* strains and the four serotypes, and this study suggest the widespread use of SNP as an antimicrobial agent for the inhibition of *S. mutans* bacteria.

## 1. Introduction

Oral health has been established as a very important condition for maintaining general health in human beings. Specifically, oral diseases represent an important challenge due to the high complexity of their components, which can influence their pathological development. Dental caries, as a multi-factorial infection, is the most common and prevalent chronic oral disease in childhood and is considered a serious worldwide public health problem [1]. *Streptococcus mutans* (*S. mutans*) has been considered the principal oral pathogen involved in the development of dental caries [2]; however, these bacteria have also been associated with other systemic diseases, such as bacteremia and infective endocarditis [3]. One of the major mechanisms that results in high cariogenicity of *S. mutans* is the ability of the bacteria to adhere to tooth surfaces. This adherence is mainly accomplished using extra-cellular polysaccharides (EPSs) derived from sucrose, but other specific microbiological characteristics related to bacterial cell wall composition might also be involved. Serologically, *S. mutans* is divided into four groups (*c*, *e*, *f,* and *k*) according to the chemical characteristics of rhamnose-glucose antigens on their cell surfaces [4]. Serotype *c* is the most prevalent serotype in the human mouth (70%–80%), followed by serotype *e* (20%), while serotypes *f* and *k* are extremely rare (5% and 2%–5%, respectively) [5,6,7]. Furthermore, specific *S. mutans* serotypes have been associated with the pathogenesis of systemic diseases such as bacteremia, infective endocarditis, and liver disease under certain conditions [8,9,10,11]. Previous studies have reported differences in the susceptibility between standard strains and clinical isolates of the mutans streptococci group, including *Streptococcus mutans*, *Streptococcus sobrinus,* and others. These differences demonstrate the need to test clinical isolates of the mutans to obtain more realistic data based on the influence of factors involved in bacterial resistance, such as patient nutrition. Studying clinical *S. mutans* isolates might be useful for investigating their pathogenesis and epidemiology as well as the prevention of dental caries [12,13,14].

Most successful protocols for the prevention and control of dental caries have been implemented [15,16]. However, the prevalence of dental caries in some countries has not diminished significantly [1,17], suggesting that the application of novel antimicrobial agents may be one of the most important priorities for the control of dental caries. An acceptable alternative to the most successful protocols is to use silver nanoparticles (SNP), which are metallic nanomaterials with high bactericidal activity against a wide variety of microorganisms [18,19]. SNP used for the prevention and treatment of dental caries could potentially control the growth and adhesion of *S. mutans* bacteria. The size of the SNP plays an important role in the antimicrobial activity of SNP against various microorganisms, including *S. mutans,* (with smaller SNP producing better antimicrobial effects). Additionally, other factors such as ion release capacity, the presence of coatings, zeta potential, and other physical and chemical properties could be involved [14,18,19]. Furthermore, SNP are toxic to human cells and this toxicity could be related to one of the previously mentioned properties [20,21]. Biocompatibility is one of the most important conditions for antimicrobians. Therefore, new and modified nanostructured metals with improved biocompatibility and antimicrobial effects are being developed through biofunctionalization with organic components [22]. Bovine serum albumin (BSA) and chitosan (CS) are two interesting organic compounds with a wide range of physiological functions and important biological properties respectively [23,24]. Albumins have several biological functions due to their ability to carry drugs as well as endogenous and exogenous substances. However, BSA also known to have a wide range of physiological functions such as the binding, transport and delivery of fatty acids, porphyrins, billirubins, steroids, and others [23,25]. CS is a non-toxic, inexpensive, and biocompatible polymer; that can be biodegradable by different hydrophilic enzymes and has important biological effects such as antimicrobial, anti-inflammatory, antioxidant, and antitumor properties [24]. Chitosan has been widely used in the regeneration of different types of tissues, especially skin and bones, and has been used in many other biomedical and pharmaceutical applications [26,27].

Although the interactions of BSA and CS with SNP have been described [25,26,27,28,29], the antimicrobial properties against clinical isolates of *S. mutans* and their serotypes of different sizes of SNPs with different coatings has not been studied in relation to active caries. The evaluation of the antimicrobial effects of different shapes and sizes of SNP coated with organic compounds against clinical strains of *S. mutans* bacteria could potentially lead to the development of therapies for the prevention and control of dental caries. One aim of the present research was to synthesize and characterize three different sizes and shapes of BSA and CS coated SNP. Another aim was to determine the antimicrobial activity of the synthesized SNP against several *S. mutans* serotypes sampled from patients with dental caries. The results of this work recommend the inclusion and wide use of BSA and CS coated SNP against the inhibition of *S. mutans* bacteria and as possible antimicrobial agents for the treatment and/or prevention of dental caries.

## 2. Results and Discussion

### 2.1. Characterization of BSA and CS Coated SNP

**Figure 1:**
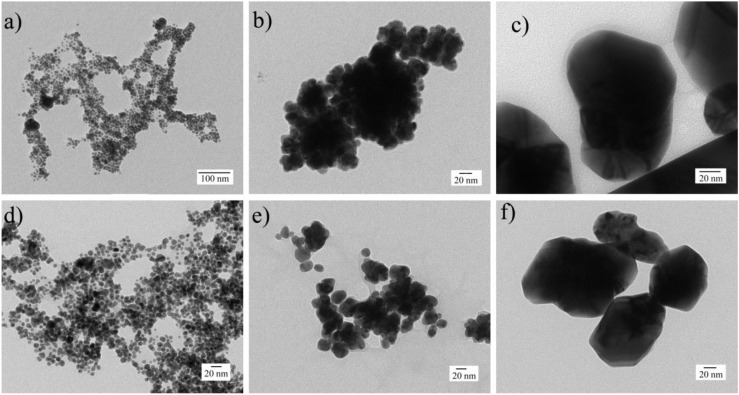
Transmission elctron microscopy (TEM) and disperion light scattering (DLS) analysis. (**a**) BSA 16.5 nm; (**b**) BSA 23.3 nm; (**c**) BSA 115.2 nm; (**d**) CS 22.5 nm; (**e**) CS 44.1 nm; and (**f**) CS 133.7 nm.

The physical, chemical, and thermal characteristics of coated SNP are shown in Table 1. Good size distributions and acceptable dispersion were found in SNP coated with BSA and CS compounds, which formed spherical and pseudospherical shapes in smaller and larger coated SNP, respectively (Figure 1). According to the dynamic light scattering (DLS) results, single narrow peaks were obtained in coated and non-coated SNP samples (16.5, 23.3, and 115.2 nm for BSA-SNP samples; 22.5, 44.1, and 133.7 nm for CS-SNP samples and 7.1, 17.4, and 87.6 nm for non-coated samples) with considerably smaller sizes for non-coated samples compared to coated SNP. The zeta potential of the larger BSA-SNP particles was negative even though the particles were larger compared to non-coated SNP samples. However, positive zeta potential values were gradually increased for CS-SNP samples with their particle size. These results suggest that the changes in the electrical charges compared to non-coated SNP are due to the presence of the BSA and CS on the particle surface, which may result from the ability of the SNP to bind to thiols and amino groups, respectively [30,31,32,33,34,35].

**Figure 2:**
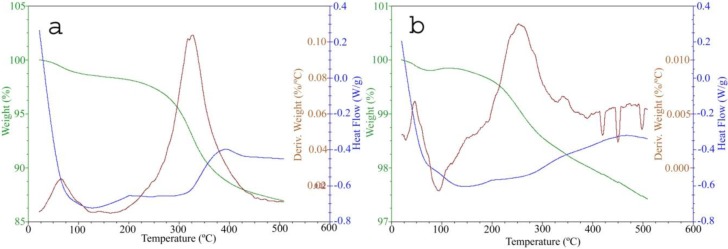
DSC-TGA analysis. (**a**) BSA 23.3 nm and; (**b**) CS 44.1 nm. Green, brown and blue lines represent TGA, DSC, and DTG values, respectively.

Thermo-gravimetric analysis (TGA) indicated more weight loss for the BSA-SNP and CS-SNP samples than the non-coated samples (Table 1, Figure 2). Figure 2a shows the TGA and differential scanning calorimetry (DSC) curves obtained from the thermal degradation of BSA at 20 °C·min^−1^ heating rate. The TGA curve shows the occurrence of three thermal events up to 500 °C. Those thermal events are associated with the dehydration and thermal degradation stages of BSA. The first endothermic peak takes place between 40 and 125 °C, with a minimum at 64 °C. Here, weight loss is attributed to water evaporation in the BSA structure. The second thermal event starts at 200 °C and finishes at 460 °C (with a minimum at 360 °C), with a corresponding weight loss of 12.5% attributed to the denaturation of BSA based on thermal degradation. The existence of considerable amounts of BSA on the SNP surface is observed in the weight loss differences, suggesting that the samples with BSA were not completely decomposed under N_2_ atmosphere due to the formation of thermally stable carbonaceous products. On the other hand, the decomposition of chitosan occurs in a single step as indicated by the DTG curves. Figure 2b shows the TG and DSC curves obtained by the thermal degradation of chitosan at a 20 °C·min^−1^ heating rate. The TGA curve shows three thermal events; however, in the DTA curve at least five thermal events, both endothermic and exothermic, are observed up to 500 °C. These thermal events are associated with the dehydration and thermal degradation processes, including the depolymerization and decomposition stages of chitosan and char combustion. The first endothermic peak takes place between 50 and 120 °C, with a minimum at 94 °C. Here, weight loss is attributed to the evaporation of water from the chitosan structure. The second thermal event starts at 190 °C and finishes at 490 °C with a corresponding weight loss of 2.5% associated with the vaporization and burning of volatile compounds coming from thermal degradation of the chitosan polymer chain structure. DSC peaks in the 200–450 °C range are attributed to the degradation of residual cross-linked chitosan. The pyrolysis of polysaccharide structures starts with the random splitting of glycosidic bonds followed by further decomposition into acetic acids, butyric acids, and a series of lower (mainly C2, C3, and C6) fatty acids [36]. The difference in the mass profiles between coated and non-coated SNP that were observed during thermal analysis suggested the presence of BSA and CS components on the surface of the SNP. Our results are consistent with previous studies that have reported the interaction of Ag^+^ ions on the SNP surface with proteins and amino acids on the BSA and free amino and hydroxyl groups on the CS molecules through steric stabilization [28,34] and electrostatic interactions [29,35,37], respectively.

### 2.2. Antibacterial Test

**Figure 3:**
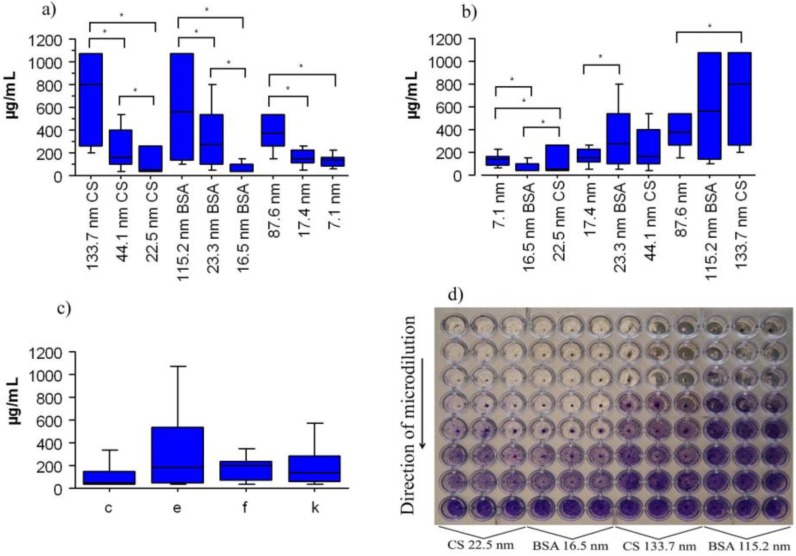
The antimicrobial activity of coated SNP against *S. mutans* in micrograms per milliliter (µg/mL). (**a**) SNP size; (**b**) coating type; (**c**) *S. mutans* serotypes; and (**d**) microdilution plaque (purple coloration shows stained bacteria). Values shown are the mean ± standard deviation. Clinical strains, 34 in number, (*c* = 20; *e* = 6; *f* = 1 and *k* = 7) were assayed. Asterisks indicate significant differences (*p* < 0.05).

**Figure 4:**
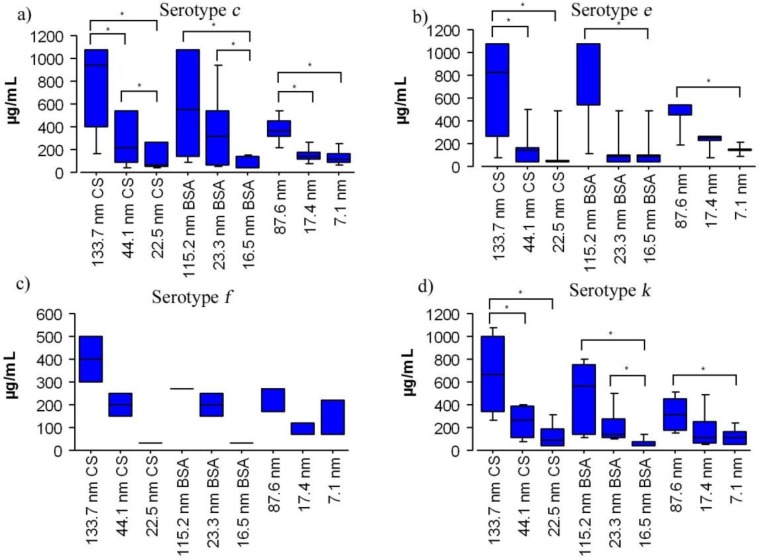
The antimicrobial activity of coated SNP agasint *S. mutans* serotypes in microgams per milliliter (µg/mL) according to serotypes and particle size. Values shown are the mean ± standard deviation. Clinical strains, 34 in number, (*c* = 20; *e* = 6; *f* = 1 and *k* = 7) were assayed. Asteriks indicate significant differences (*p* < 0.05).

**Figure 5:**
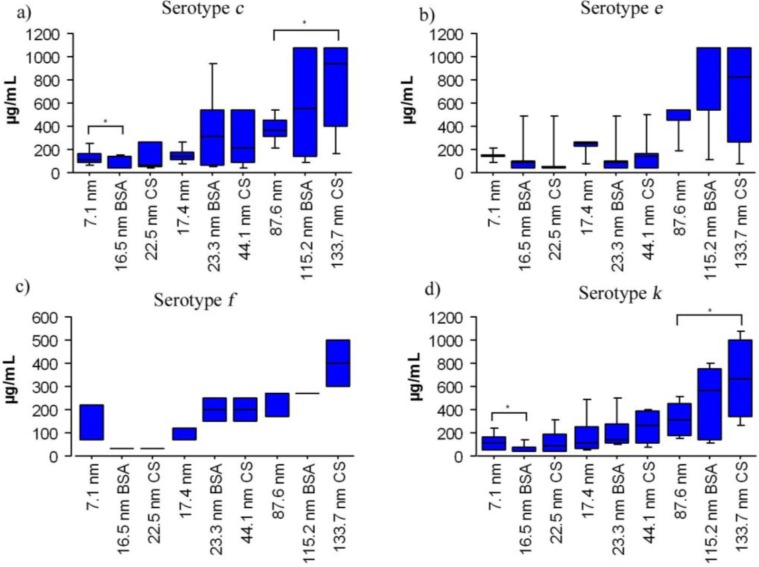
The antimicrobial activity of different sizes of SNP with either no coating, a BSA coating or a CS coating in micrograms per milliliter (µg/mL). Values shown are the mean ± standard deviation. Clinical strains, 34 in number, (*c* = 20; *e* = 6; *f* = 1, and *k* = 7) were assayed. Asterisks indicate significant difference (*p* < 0.05).

Results of the antibacterial assays are shown in Figure 3. The three different sizes of BSA-SNP and CS-SNP samples showed antimicrobial activity against clinical stocks of *S. mutans* serotypes *c*, *e*, *f,* and *k* with significant differences between the size of the SNP and the type of coating. The antibacterial differences of BSA-SNP and CS-SNP samples were higher when the size of the particles was decreased (Figure 3a,d), but the type of coating of the SNP also influenced the antimicrobial effect (Figure 3b). The 16.5 nm BSA-SNP samples (83.0 ± 97 µg/mL) had better antimicrobial effect compared to the 22.5 nm CS-SNP samples (127.3 ± 123 µg/mL) (Figure 3b, Figure 5a,d). Serotype *f* was the most sensitive to SNP (175.7 ± 118 µg/mL) followed by serotypes *k* (266 ± 255 µg/mL), *c* (317.9 ± 316 µg/mL) and, the most resistant serotype, *e* (348.7 ± 339 µg/mL) (Figure 3c). However, there were no significant differences between serotypes (*p* > 0.05). This result suggests that SNP have the potential to inhibit the growth of any *S. mutans* serotype, including those serotypes involved in bacteremia, infective endocarditis, and liver disease [8,10,11]. MIC results of serotypes exposed to coated and non-coated SNP samples indicated similar significant differences between different sized particles (*p* < 0.05) with better bactericidal activities for smaller nanoparticles (Figure 4).

The antimicrobial effects of different SNP coatings on different serotypes are shown in Figure 5. There were significant differences between the MICs of BSA and CS coatings for particles of similar size in serotype *c* (BSA 22.9 nm and CS 29.7 nm). The smaller BSA-SNP samples had better inhibitory effects (84.6 ± 98 µg/mL) compared to smaller CS-SNP samples (130.2 ± 124 µg/mL, Figure 5a). The antimicrobial effect of the smaller BSA-SNP samples (22.9 nm) was also better in serotypes *e* (91.6 ± 103 µg/mL), *f* (33.4 ± 0 µg/mL), and *k* (83.1 ± 97 µg/mL) than the smaller CS-SNP samples (22.5 nm) on the same serotypes (130.2 ± 124, 142.4 ± 138, 33.4 ± 0, and 127.3 ± 123 µg/mL, respectively); however, no significant differences were found (*p* > 0.05). Additionally, in an attempt to explore the mechanism of the antibacterial effect of coated SNP on *S. mutans*, statistical comparisons of SNP size and coating were made. No significant differences were found between pairs of *S. mutans* serotypes (*c/e*, *c/f*, *c/k*, *e/f*, *e/k,* and *f/k*) for either BSA or CS-SNP samples (BSA 16.5, BSA 23.3, BSA 115.2, CS 22.5, CS 44.1, and CS 133.7) indicating they are affected in a similar manner by both types of coated SNP; in other words, it is possible that all *S. mutans* serotypes can be inhibited by coated SNP penetration of the cell wall despite differences in structure among serotypes. Additionally, the large standard deviation values, for MICs of the largest SNP sizes (CS 133.7 nm, BSA 115.2 nm, and 87.6 nm, respectively), might be caused by demographic, nutritional, immunological, and microbiological variations of each individual patient from which the clinical isolates were sampled. These variations may include diet, antimicrobial agents, oral habits, particular host responses, cariogenicity levels, and bacterial resistant among others. It is possible that serotypes with similar biochemical characteristics may show different antimicrobial responses; however, this topic should be further evaluated by testing additional antimicrobial agents and through other microbiological techniques.

Microbiological differences among serotypes are based in part on the chemical composition of the specific polymeric component of the cell wall [4,7]. These components could modulate the resistant or sensitivity to SNP; however, few studies have evaluated this effect on *S. mutans* serotypes. One study in 2012 reported differences in the antimicrobial effect of silver nanoparticles (without coatings) on different *S. mutans* serotypes concluding that the sensitivity of *S. mutans* depends on particle size and the type of serotype, with some serotypes being more sensitive to the antimicrobial activity of SNP [31] and suggesting SNP is a selective antimicrobial agent. Our findings suggest that the broad inhibition on clinical isolates of *S. mutans* serotypes depends on combination of small SNP particle sizes and the presence of a specific coating on the SNP surface. The antibacterial action might be due to the high surface areas on small particles [38] and the binding capacity of coated SNP on the cell membranes [39,40]. This action is probably mediated by electrostatic interactions and by interactions with the adhesion system (EPSs) of the bacteria cell membrane, which allows for strong linkages with the amino, hydroxyl and thiols groups of CS and BSA [39,41]. On the other hand, larger CS coated SNP samples (133.7 nm) had the worst antimicrobial activity compared to BSA coated SNP samples (115.2 nm) and similarly sized non-coated SNP samples (87.6 nm). It is possible that both the larger size and the presence of CS compound on the surface of the SNP are the principal factors involved in the low antimicrobial inhibition capacity of the 133.7 nm CS coated SNP. This low capacity may be the result of the reduced surface area compared to BSA-SNP and non-coated SNP and the larger size of the chitosan molecule, which may prevent the CS coated SNP from entering the cell and exerting its antimicrobial activity [38]. Our results are in agreement with previous reports that the antibacterial activity of coated SNP against different types of microorganisms, including *S. mutans* bacteria, is associated with the particle size and the presence and type of coating on the SNP surface. However, previous reports only tested SNP on standard reference strains [37,38,42]. In addition, the microdilution method used in this study included a representative amount of sucrose (2%) and this characteristic of the BHI broth improves the cariogenic potential in the metabolism of *S. mutans* bacteria, allowing for more objective and realistic data based on the presence of sugar in our daily diet. In future studies, a larger number of clinical isolates from each *S. mutans* serotype should be included for a better understanding of coated SNP on *S. mutans* bacteria.

### 2.3. Scanning Electron Microscopy

**Figure 6:**
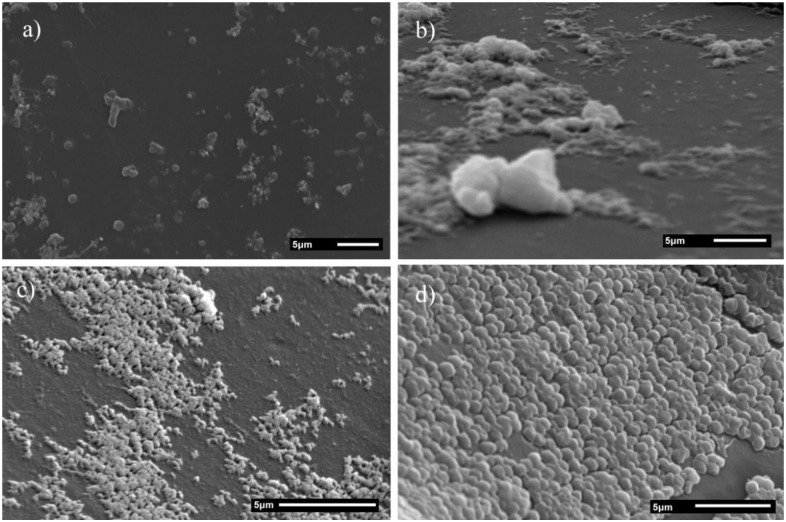
SEM micrographs of *S. mutans* (serotype *c*) with coated SNP. (**a**) 10.7 nm; (**b**) BSA 22.9 nm; (**c**) CS 29.7 nm; and (**d**) control (without SNP).

SEM micrographs of *S. mutans* cells (serotype *c*) exposed to coated and non-coated SNP are shown in Figure 6. Growth inhibition areas are observed in well surfaces with *S. mutans* cells and SNP. Binding alterations among cell-cell, cell-surface, and no morphological changes around cell membranes can be observed in wells exposed to coated and non-coated SNP samples. Additionally, BSA-SNP and CS-SNP samples had similar bacterial growth inhibition zones compared to non-coated SNP, but there was less bacterial growth and non-adhered bacterial cells were also evident. The mass of *S. mutans* bacteria in Figure 3b is the result of cell adhesion, representing an isolated example of bacterial aggregation by the production of extracellular polysaccharide, consisting of soluble and insoluble glucans that are beneficial to survival [43,44]. The active mechanism of the coated SNP might be influenced by the mechanical and chemical affinities for components of the *S. mutans* cells: polysaccharides, lipoteichoic and teichoic acids, and dextrans, among others, which form a strong bond with the BSA and CS compounds, improving surface interactions between the bacteria cell membrane and the surface of coated SNP [43,44]. The results suggest that BSA and CS components on the SNP surfaces might help to increase interactions directly between SNP and the cell membranes of *S. mutans,* therefore promoting antibacterial activity. Moreover, additional in vitro and in vivo studies should be conducted to understand the antibacterial mechanism of BSA and CS coated SNP. We explored the mechanisms of adhesion inhibition using clinical isolates of *S. mutans* bacteria and future studies should be focused on analyzing the effects of SNP on the adhesion of *S. mutans* strains to dental enamel samples or other types of surfaces.

The fabrication, preparation, design, application and evaluation of novel antimicrobial agents using metallic nanomaterials, most importantly Ag, should be the focus of research to produce more sustainable and safe compounds by minimizing the citotoxicity level associated with the physical and chemical properties of SNP. Recent studies have evaluated the relationship of Ag ions and their cytotoxic effect in eukaryotic and prokaryotic cells. These studies concluded that Ag ions show different cytotoxic responses between eukaryotic and prokaryotic cells. Higher concentrations of Ag might produce comparable cytotoxic damage among eukaryotic cells because of their larger size, higher structure and more complex metabolism compared to prokaryotic cells [45,46]. Although our study only evaluated the antimicrobial effect of coated SNP against clinical isolates of *S. mutans* and its serotypes, it is possible that coated SNP may only affect bacterial cells and not humans cells, thereby providing a good bactericidal activity with minimal cytotoxic effects. Notwithstanding, BSA and CS coated SNP could be widely used to control *S. mutans* bacteria in the prevention and treatment of dental caries through the use of solutions applied directly to the surface of the tooth enamel, or by inclusion in toothpastes, mouthwashes, chewing gums, and the bristles of toothbrushes. More in vitro and in vivo studies are needed to evaluate SNP biocompatibility and to guarantee their safe use for biomedical and dental applications.

## 3. Materials and Methods

### 3.1. Materials and Reagents

Silver nitrate (AgNO_3_, CTR Scientific, Monterrey, Nuevo León, México), bovine serum albumin (BSA, Sigma-Aldrich, Saint Lois, MO, USA), chitosan (low molecular weight 85%, Sigma-Aldrich), gallic acid (C_7_H_6_O_5_, Sigma-Aldrich, Saint Lois, MO, USA), acetic acid (C_2_H_4_O_2_, 99.7%, J.T. Baker, Center Valley, PA, USA), and brain-heart infusion broth (BHI^™^, DIFCO Laboratories, Detroit, MI, USA), were used and stored according to manufacturer’s recommendations. All reagents used were of analytical grade. Deionized water (18 M) was prepared using a Milli-Q Biocel Purification System (Merck Millipore Corporation, Billerica, MA, USA).

### 3.2. Silver Nanoparticles Preparation

The synthesis of the three different sizes of coated SNP using BSA and CS compounds was performed as previously reported by Espinosa-Cristobal et al., 2015 [30]. All solutions started with a 0.01 M AgNO_3_ solution and, under magnetic stirring, 10 mL of deionized water with 0.1–0.5 g of gallic acid (first and second sized samples, respectively) was added to 100 mL Ag^+^ solution for each SNP sample. After the addition of gallic acid, the pH value of the solution was immediately adjusted (for the first sample the pH was raised to 11 with 1.0 M NaOH and for the second sample the pH was raised to 10 with NH_4_OH) [31]. After that, the first and second suspensions were agglomerated by the addition of nitric acid (63%) until pH 1.5 was reached. Suspensions were vacuum filtered through a nitrocellulose filter (Millipore, 0.1 µm pore, Darmstadt, Germany) in a vacuum filter flask. So, deionized water was repeatedly used to wash the SNP samples on the filter. Once pH 8 was reached, membranes with SNP were suspended to a metered volume. Additionally, a third sample of SNP was also prepared. This preparation was similar to the first and second SNP samples; however, after the addition of gallic acid the solution was irradiated with UV light (254 nm, 15 W) for 30 min, and heated for 30 min at 80 °C. Finally, the solution was centrifuged at 9000 rpm for 10 minutes (Biofuge, Heraeus, PA, USA), washed three times, and re-suspended in a metered volume with deionized water.

### 3.3. Preparation of BSA Coated Silver Nanoparticles

1% of a BSA solution (wt/vol) was prepared using deionized water and mixed in 50 mL tubes with different sizes of SNP, respectively. For each SNP size, the proportions (SNP/BSA) 7:1 for the first sample; 14:1 for the second sample; and 7:1 for the third sample were used. Each solution was mixed for 12 h on a magnetic stir plate at ambient temperature.

### 3.4. Preparation of CS Coated Silver Nanoparticles

CS solutions with different concentrations of acetic acid were prepared by dissolving purified CS in 1%, 0.5%, and 0.25% acetic acid solutions to reach a concentration of 1% (wt/vol) and these solutions were magnetically stirred for 12 h until the solutions were transparent. To obtain CS coated small SNP (first sample) a solution containing 0.5% of acetic acid with 1% of dissolved CS was mixed with the first size of SNP in a proportion of 56:1 (SNP/CS); for the second size of CS coated SNP a solution containing 0.5% of acetic acid with 1% of CS was combined with the second size of SNP in a proportion of 28:1; and finally, for the largest size of CS coated SNP, an acetic acid solution of 0.25% dissolved with 1% of CS was combined with the third size of SNP in a proportion of 28:1. Each solution was mixed for 12 h on a magnetic stir plate at ambient temperature.

### 3.5. Characterization

The BSA and CS coated SNP were characterized using dynamic light scattering (DLS, Malvern Zetasizer Nano ZS, Malvern, Worcestershire, UK) and transmission electron microscopy (TEM, JEOL JEM-1230, Tokyo, Japan) at an accelerating voltage of 100 kV. Thermo-gravimetric analysis (TGA) and differential scanning calorimetry (DSC) analysis were performed using a DSC calorimeter (SDT Q600, TA Instruments, New Castle, DE, USA) with a constant heating rate of 20 °C/min from room temperature to 510 °C under a nitrogen atmosphere.

### 3.6. Identification of S. mutans Serotypes Using PCR

Thirty-four clinical isolates stocks of *S. mutans* (serotypes *c* = 20; *e* = 6; *f* = 1, and *k* = 7) were provided by the Master´s Degree in Dental Science at San Luis Potosi University, Mexico. The isolates were collected from saliva samples of 3 to 6 year old children with active caries and without fillings. *S. mutans* samples were grown in MSB medium (Mitis Salivarius Agar and Bacitracin) supplemented with 2% sucrose and 1% of potassium tellurite. Agar plates were incubated at 37 °C for 48 h in a CO_2_ atmosphere and for an additional 48 h at room temperature. Colony forming units (CFU) and *S. mutans* strain were macroscopically identified using a Stereoscopic Microscope (Olympus, SD-ILK, Tokyo, Japan). Tubes containing bacterial pellets from one *S. mutans* colony grown in BHI broth for 24 h at 37 °C were washed in 1 mL of PBS (pH 7.4), re-suspended in 200 μL of cell lysis buffer (1.0% Triton X-100, 20 mM Tris–HCl, 2 mM EDTA, pH 8.0) and incubated at 85 °C for 10 min. Next, 100 μL of mutanolysin at 200 Units/mL (Sigma, St. Louis, MO, USA) was added and the sample was incubated at 50 °C for 1 h, followed by treatment with 80 μL of nuclei cell lysis solution at 80 °C for 10 min. Then, 60 μL of protein solution were precipitated and removed by centrifugation at 13,000 rpm for 10 min). DNA was purified by extraction with phenol-chloroform-isoamyl alcohol (25:24:1, vol/vol; Invitrogen, Carlsbad, CA, USA) and was further precipitated with isopropanol. The extracted DNA was dissolved in 100 μL of DNA hydration solution, which was used for the PCR (Polymerase Chain Reaction) assays. All solutions were used according to the manufacturer’s instructions (Puregene DNA isolation Kit, Gentra Systems, Minneapolis, MN, USA) for Gram-positive bacteria. PCR assays were performed in a 25 μL reaction mixture containing 0.5 U Taq polymerase (Roche, Mannheim, Germany), 0.5 μM of gene specific primers, 5 μL (10 ng/μL of DNA template) and 1.5 mM of MgCl_2_, following the manufacturers protocol. The PCR reaction was performed in a thermal cycler (iCycler, BIO-RAD laboratories, Hercules, CA, USA) with the following cycling parameters: an initial denaturation at 98 °C for 3 min, 30 cycles of denaturation at 98 °C for 10 s, annealing at 70 °C for 1 min, extension at 70 °C for 1 min and a final extension at 70 °C for 4 min. The positive controls used for each DNA serotypes were GS5 and NG71 for serotype *c*, LOMA13 for serotype *e*, OMZ175 for serotype *f* and FT1 for serotype *k*. The primers used in this study to identify *S. mutans* and *S. mutans* serotypes were reported by Nakano et al., 2004 [7] (serotype *k*, size 300 bp [CEFK-F: ATTCCCGCCGTTGGACCATTCC; and K-R: CCAATGTGATTCATCCCATCAC]), Shibata et al., 2003 [32] (serotype *c*, size 727 bp [SC-F: CGGAGTGCTTTTTACAAGTGCTGG; and SC-R: AACCACGGCCAGCAAACCCTTTAT]; serotype *e*, size 517 bp [SE-F: CCTGCTTTTCAAGTACCTTTCGCC; and SE-R: CTGCTTGCCAAGCCCTACTAGAAA]; serotype *f*, size 316 bp [SF-F: CCCACAATTGGCTTCAAGAGGAGA; and SF-R: TGCGAAACCATAAGCATAGCGAGG]) and Hoshino et al., 2004 [33] (*S. mutans*, size 433 bp [MKD-F: GGCACCACAACATTGGGAAGCTCAGTT; and MKD-R: GGAATGGCCGCTAAGTCAACAGGAT]). Electrophoresis assay was used to analyze the PCR products through 2% of agarose gels including a Tris-acetate-EDTA buffer and a 100-bp DNA ladder (New England Biolab, Beverly, MA, USA) as molecular size marker. An ethidium bromide solution (0.5 μg/mL) was used to stain each agarose gel. Images of each gel were obtained under UV illumination (ChemiDoc, BIO-RAD laboratories, Hercules, CA, USA) identifying and classifying to the *Streptococcus mutans* bacteria and its four serotypes (*c*, *e*, *f*, and *k*).

### 3.7. Antibacterial Assay

The antibacterial testing method used in this work was reported by Espinosa-Cristobal et al., [30,31]. *S. mutans* strains subclassified according to serotypes (as previously identified PCR) were cultured in brain-heart infusion broth (BHI) for 18 h at 37 °C. The distribution of serotypes obtained was *c* = 20, *e* = 6, *f* = 1, and *k* = 7. A microdilution plate assay was used and SNP sample was mixed with BHI medium containing 2% sucrose, and inoculated with 6 × 10^5^ CFU/mL of each clinical isolate strains. After 24 h at 37 °C of incubation, minimum inhibitory concentrations (MIC) were determined based on turbidity (bacterial growth). To confirm the MIC, all wells were washed with PBS (phosphate buffered solution), fixed in 0.1% glutaraldehyde for 5 min and stained with 0.5% crystal violet. The MIC was considered the minimum necessary concentration to promote antimicrobial activity in the last well that presented coloration. Purple coloration indicated the presence of *S. mutans* bacteria (Figure 3d). Both turbidity and coloration tests were performed in triplicate to determine MICs. Scanning electron microscopy (SEM, JEOL JSM-6510, at an accelerating voltage of 20 kV, Akishima, Tokyo, Japan) was used to analyze the antimicrobial activity of coated SNP samples on *S. mutans* cells on wells from microdilution plates.

### 3.8. Statistical Analysis

All data were expressed as the mean ± standard deviation. Significant differences between SNP samples and *S. mutans* serotypes groups were analyzed by Mann Whitney U test for non-parametric values (StatView software, SAS Institute Inc., v5.0.1, Cary, NC, USA). Samples were considered significantly different when *p* < 0.05.

## 4. Conclusions

The BSA and CS coated SNP with different sizes and shapes were successfully prepared and characterized. All of the treatments had antibacterial activity against clinical stocks of *S. mutans* bacteria. Higher antimicrobial activity was associated with smaller SNP and the presence of BSA coating. All samples of serotypes *c*, *e*, *f,* and *k* had similar sensitivity levels to the three different sizes and coatings of SNP; however, serotypes *c* and *e* were considered the most resistant, although this difference was not statistically significant. This work indicates that BSA and CS coated SNP have strong antimicrobial effects against *S. mutans* bacteria including the serotypes *c*, *e*, *f,* and *k*, suggesting the potential for biomedical uses. According to our knowledge, this is the first study to determine the antimicrobial activity of three different sizes and shapes of BSA and CS coated SNP against clinically isolated stocks of *S. mutans* bacteria across four different serotypes.

## Figures and Tables

**Table 1:** Physical and thermal properties of the BSA and CS coated SNP.

SNP Sample (nm)	Diameter DLS (nm)	Zeta Potential (mV ± ZD)	Residues TGA (%)	Total Weight Loss TGA (%)
7.1	7.1	−48.4 ± 6.9	98.5	1.5
17.4	17.4	−52.6 ± 8.5	98.2	1.7
87.6	87.6	−55.7 ± 9.9	97.8	2.1
BSA 16.5	16.5	−13.5 ± 4.7	96.3	3.6
BSA 23.3	23.3	−44.0 ± 6.9	86.9	13.0
BSA 115.2	115.2	−32.7 ± 6.3	96.7	3.2
CS 22.5	22.5	37.9 ± 13.0	67.3	32.6
CS 44.1	44.1	48.3 ± 7.9	97.5	2.4
CS 133.7	133.7	52.0 ± 5.4	95.9	4.1

## Abbreviations

The following abbreviations are used in this manuscript:

µg/mL	microgram per milliliter
BSA	Bovine serum albumin
CFU	colony forming units
CS	Chitosan
DLS	Dynamic light scattering
DSC	Differential scanning calorimetry
MIC	Minimum inhibitory concentration
*S. mutans*	*Streptococcus mutans*
SEM	Scanning electron microscopy
SNP	Silver nanoparticles
TEM	Transmission electron microscopy
TGA	Thermo-gravimetric analysis
